# Optimal Low-Density Lipoprotein Cholesterol Levels in Adults Without Diabetes Mellitus: A Nationwide Population-Based Study Including More Than 4 Million Individuals From South Korea

**DOI:** 10.3389/fcvm.2021.812416

**Published:** 2022-01-20

**Authors:** Ji Hye Huh, Sang Wook Park, Tae-Hwa Go, Dae Ryong Kang, Sang-Hak Lee, Jang-Young Kim

**Affiliations:** ^1^Division of Endocrinology and Metabolism, Department of Internal Medicine, Hallym University Sacred Heart Hospital, Anyang, South Korea; ^2^Division of Cardiology, Department of Internal Medicine, Yonsei University Wonju College of Medicine, Wonju, South Korea; ^3^Department of Biostatistics, Yonsei University Wonju College of Medicine, Wonju, South Korea; ^4^Division of Cardiology, Department of Internal Medicine, Yonsei University College of Medicine, Severance Hospital, Seoul, South Korea

**Keywords:** low-density lipoprotein cholesterol, impaired fasting glucose, cardiovascular disease, mortality, glucose level

## Abstract

**Background:**

Although the strong association between low-density lipoprotein cholesterol (LDL-C) and cardiovascular disease (CVD) is well-known, the threshold LDL-C level at which the risk of CVD begins to increase in individuals without diabetes mellitus (DM) remains unknown. We aimed to evaluate the association between incident CVD and serum LDL-C levels with or without statin use in individuals without DM.

**Methods:**

We identified 4,182,117 individuals without previous CVD who underwent a health screening examination in 2009 and 2011 from the Korean National Health Insurance Cohort database. The primary endpoint was a composite of cardiovascular deaths, myocardial infarction (MI) cases, and ischemic stroke cases.

**Results:**

During the median follow-up of 6 years, there were 51,961 CVD events that included 17,392 MI cases, 33,779 ischemic stroke cases, and 2,039 cardiovascular deaths. The LDL-C levels that were associated with an increased risk of CVD were ≥100 mg/dL in non-statin users and ≥130 mg/dL in statin users. However, even in individuals with lower LDL-C levels, all those with fasting plasma glucose (FPG) levels ≥110 mg/dL had a significantly higher risk of CVD.

**Conclusions:**

We demonstrated that LDL-C levels ≥100 mg/dL were correlated with an increased risk of CVD in individuals without DM and a history of CVD. We found that a glucose, cholesterol interaction increased CVD risk, and modestly elevated FPG levels (110–125 mg/dL) were associated with a higher CVD risk even in individuals with well-controlled LDL-C levels.

## Introduction

Cardiovascular disease (CVD) is the leading cause of mortality globally and accounted for 31.4% of all deaths in 2012. In developed countries, age-adjusted cardiovascular mortality rates are declining; however, CVD remains the leading cause of mortality due to the rapid aging of the population (1). Low-density lipoprotein cholesterol (LDL-C) is considered to be a major causative factor in the development of atherosclerotic CVD (ASCVD). Numerous studies have robustly demonstrated that reductions in plasma LDL-C concentrations by lipid-lowering agents are strongly associated with the reduced risk of incident CVD (2, 3). Most current guidelines include the LDL-C level as a primary indicator for initiating and adjusting lipid-lowering interventions. However, these guidelines were set based on data from randomized controlled trials that investigated specific LDL-C targets for adjustments in the statin dose or from a small group of highly selected studies. Few longitudinal large-scale epidemiological studies have investigated the optimal LDL-C range for the lowest risk of CVD, especially in individuals without a prior history of CVD and diabetes mellitus (DM).

Hyperglycemia and a high LDL-C level have been considered major risk factors for CVD (4). Patients with DM have a 2–4 times higher risk of CVD and cardiovascular mortality than the general population. Individuals with DM have a higher risk of the presence of small dense LDL particles than those without DM, even at identical plasma LDL-C levels. Therefore, the current guidelines regarding lipid management recommend the use of statins according to the LDL-C levels to prevent CVD in most patients with DM. However, considering the direct effect of high plasma glucose levels on CVD independent of LDL-C levels, optimal LDL-C goals for primary prevention may differ according to the glucose levels, even in individuals without DM. Nevertheless, trials that investigated the LDL-C target for the primary prevention of CVD have been performed among both patients with DM and individuals without DM (5), as well as among different ethnic groups (6). Also, these previous studies did not consider the possible synergistic effects between higher plasma glucose levels and higher LDL-C levels.

In this study, we aimed to identify the optimal LDL-C levels associated with the lowest risk of CVD incidence in individuals without DM. We also evaluated whether fasting plasma glucose (FPG) levels magnify the risk of CVD associated with LDL-C levels in individuals without DM. Moreover, we classified the study population into statin users and non-users to investigate the optimal LDL-C level for the primary prevention of CVD in each glycemic status classification. For this analysis, we used large-scale nationwide cohort data from the Korean National Health Insurance System (NHIS) database, which represents the entire Korean population.

## Methods

### Study Population

In our cohort study, we used data from the NHIS, which is a government program that was implemented in 2002 and includes data on ~98% of the Korean population. Participants are entitled to a general health screening program every 2 years. Standardized self-reported questionnaires on medical history, lifestyle habits, anthropometric and blood pressure measurements, and regular laboratory tests using blood and urine samples are all part of the screening (7). Our research-specific database included data from 2009 to 2011 for participants aged 19–69 years who underwent at least two general health screening programs during this period. To exclude participants who experienced myocardial infarction (MI) or ischemic stroke, those who had the following International Classification of Diseases, 10th Revision (ICD-10) codes (as the main diagnosis or sub-diagnosis at baseline) were not included: I21, I22, I23, I63, or I64. We excluded those who were aged <40 years in 2009 and did not participate in a general health screening program in 2009. Thus, 4,709,862 participants were eligible for this analysis (8). Participants who had missing variables regarding cholesterol and fasting glucose levels and were already diagnosed with DM (FPG level of ≥126 mg/dL or at least one claim for the prescription of hypoglycemic drugs per year, including insulin, under the ICD-10 codes E11–14) at baseline were excluded (9). We also excluded those who had a serum glucose level of 70 mg/dL and died before 2014 or those who died due to unknown causes. Finally, 4,182,117 participants were included at baseline (Supplementary File 1). This study was approved by the Institutional Review Board of Yonsei University Wonju College of Medicine, Republic of Korea (no. CR318356). As the study was conducted using anonymous and de-identified data, informed consent from the participants was not obtained.

### Measurements and Definitions

The NHIS data included sex, age, body mass index (BMI), height, weight, waist circumference, blood pressure, and lifestyle-related behaviors, such as the frequency of alcohol consumption per week, smoking status, and regular exercise. Regular exercise was defined as performing more than 30 min of moderate physical activity at least five times per week or more than 20 min of strenuous physical activity at least three times per week. The income level was dichotomized at the lowest 25%. Blood samples for measuring serum glucose, creatinine, and lipid levels were drawn after an overnight fast. Blood samples for measuring total cholesterol, high-density lipoprotein cholesterol (HDL-C), triglyceride (TG), and FPG levels were obtained at the health examinations after the participants fasted for at least 8 h. LDL-C levels were calculated using the Friedewald formula: LDL-C = total cholesterol – HDL-C – (TG/5). Hypertension was defined as a systolic/diastolic blood pressure of ≥140/90 mm Hg or at least one claim for antihypertensive medication prescriptions per year under the ICD-10 codes I10–I15. We defined a statin user as a person who had been prescribed statins in 2009–2011.

### Study Outcomes and Follow-Up

Participants who received two or more health screenings between 2009 and 2011 and were evaluated for primary outcomes during the follow-up period from 2014 to 2017 were included in the current study. During the follow-up period, the primary endpoint was cardiovascular events, which were described as a composite of incident cardiovascular deaths, MI cases, and ischemic stroke cases. To minimize the influence of possible “reverse causation,” we excluded participants with cardiovascular events that occurred within 3 years after baseline measurements. The ICD-10 codes were used for the diagnoses. MI was determined by recording the ICD-10 codes I21 or I22 at least twice during hospitalization for at least 4 days. Ischemic stroke was diagnosed based on the ICD-10 codes I63 or I64 that were registered during a 4-day hospital stay with claims for brain magnetic resonance imaging or brain computerized tomography (10). The Korea National Statistical Office provided nationwide death certificate data for the follow-up analyses of cardiovascular deaths. The research was deemed complete if the participants' cardiovascular events occurred or the end of the follow-up period, whichever occurred first.

### Statistical Analysis

For each group, the continuous variables are presented as means and standard deviations, and the categorical variables are presented as frequencies and percentages. The participants were classified into six groups according to the following plasma LDL-C concentrations at baseline: <70, 70–99, 100–129, 130–159, 160–189, and ≥190 mg/dL. The hazard ratios (HRs) and 95% confidence intervals (CIs) for incident CVD according to the categories of LDL-C were obtained using multivariable Cox proportional hazard models using the 70–99 mg/dL category as the reference after adjusting for age, sex, BMI, smoking status, alcohol consumption, regular exercise, income, and hypertension. We also investigated the risk of CVD according to LDL-C categories within the FPG strata. We analyzed the data using SAS version 9.4 (SAS Institute, Inc., Cary, NC) and R version 3.5.1 (R Foundation for Statistical Computing, Vienna, Austria).

## Results

### Baseline Characteristics of the Participants

The NHIS data of a total of 4,182,117 participants from 2009 to 2011 were analyzed. The mean age of the participants was 51.1 years and 2,037,288 (48.7%) participants were male. Overall, 16.7% of the participants used statins. Table 1 summarizes the baseline characteristics of the cohort groups according to the baseline LDL-C concentrations (<70, 70–99, 100–129, 130–159, 160–189, and ≥190 mg/dL). The proportion of male participants gradually increased from the lowest to the highest LDL-C categories. Participants in the highest LDL-C category (LDL-C levels ≥190 mg/dL) tended to be older, have a higher BMI, and were more likely to take statins than those in the other LDL-C categories. Patients with a low LDL-C level were more likely to be female, current smokers, and heavy drinkers. The HDL-C levels and blood pressure measurements were similar across the six LDL-C categories.

**Table 1:** Baseline characteristics of participants according to LDL-C concentrations.

**Characteristics**	**Overall**	**LDL-C (mg/dL)**						^*^P for trend
		**<70**	**70–99**	**100–129**	**130–159**	**160–189**	**≥190**	
*N*	4,182,117	158,167	954,421	1,710,682	1,031,991	274,485	52,371	
Age (years)	51.1 (7.9)	50.6 (8.2)	50.2 (8)	50.9 (7.9)	51.9 (7.7)	52.6 (7.6)	53.0 (7.5)	<0.05
BMI (kg/m^2^)	23.9 (2.8)	23.5 (3)	23.4 (2.9)	23.8 (2.8)	24.2 (2.8)	24.4 (2.7)	24.6 (2.8)	<0.05
Sex (male)	2,037,288 (48.7)	53,001 (33.5)	437,295 (45.8)	831,486 (48.6)	529,958 (51.4)	121,073 (44.1)	20,235 (38.6)	<0.05
Systolic BP (mmHg)	122.6 (12.5)	124.2 (13.2)	121.9 (12.7)	122.3 (12.4)	123.2 (12.3)	123.7 (12.3)	124.2 (12.7)	<0.05
Diastolic BP (mmHg)	76.7 (8.3)	77.8 (8.8)	76.3 (8.5)	76.6 (8.3)	77 (8.2)	77.3 (8.2)	77.5 (8.4)	<0.05
eGFR (ml/min/1.73 m^2^)	85.4 (19.5)	87 (19.7)	86.7 (19.6)	85.5 (19.5)	84.3 (19.3)	83.6 (18.9)	83.3 (18.4)	<0.05
Fasting plasma glucose (mg/dl)	94.3 (9.7)	95.2 (10.6)	93.7 (9.8)	94.1 (9.6)	94.8 (9.6)	95.6 (9.7)	96.1 (9.9)	<0.05
Total cholesterol (mg/dl)	199.8 (31.7)	152.4 (25.6)	170.1 (18)	195.3 (16.3)	223.2 (15.6)	252.8 (15.7)	290.0 (26.0)	<0.05
HDL-C (mg/dl)	55.8 (17.7)	55.6 (19.8)	56.3 (17.8)	55.8 (17.5)	55.3 (17.5)	55.3 (17.9)	55.7 (20.1)	<0.05
Triglycerides (mg/dl)	132.8 [77.9]	194.7 [156.7]	132.1 [87.8]	126.8 [68.6]	131.7 [62.6]	138.5 (62.0)	148.5 (75.3)	<0.05
Smoking status (%)								<0.05
Never smoker	2,614,379 (62.5)	77,620 (49.1)	577,272 (60.5)	1,076,249 (62.9)	664,294 (64.4)	182,791 (66.6)	36,153 (69.0)	
Former smoker	676,404 (16.2)	27,934 (17.7)	156,145 (16.4)	283,181 (16.6)	164,072 (15.9)	38,842 (14.2)	6,230 (11.9)	
Current smoker	891,125 (21.3)	52,604 (33.3)	220,956 (23.2)	351,155 (20.5)	203,582 (19.7)	52,840 (19.3)	9,988 (19.1)	
Alcohol consumption								<0.05
≤ 2 days/week	3,609,708 (86.3)	114,502 (72.4)	794,956 (83.3)	1,485,657 (86.9)	918,232 (89)	248,541 (90.6)	47,820 (91.3)	
3–4 days/week	405,491 (9.7)	27,970 (17.7)	110,844 (11.6)	161,468 (9.4)	83,063 (8.1)	18,874 (6.9)	3,272 (6.3)	
≥ 5 days/week	166,669 (4.0)	15,684 (9.9)	48,568 (5.1)	63,444 (3.7)	30,641 (3)	7,056 (2.6)	1,276 (2.4)	
Income (lower 25%)	870,665 (21.1)	32,854 (21.0)	201,380 (21.3)	356,338 (21.1)	211,823 (20.8)	56,917 (21.0)	11,353 (21.9)	<0.05
Regular exercise (%)	1,434,239 (34.3)	53,243 (33.7)	325,431 (34.1)	591,656 (34.6)	354,645 (34.4)	92,432 (33.7)	16,832 (32.1)	<0.05
Hypertension (%)	1,474,782 (35.3)	67,978 (43)	337,057 (35.3)	594,109 (34.7)	362,237 (35.1)	94,897 (34.6)	18,504 (35.3)	<0.05
Medication for statin (%)	699,882 (16.7)	32,525 (20.6)	116,728 (12.2)	196,774 (11.5)	210,196 (20.4)	81,596 (29.7)	15,599 (29.8)	<0.05

*Data are mean (standard deviation), median [interquartile range], or percentage*.

*BMI, body mass index; BP, blood pressure; HDL-C, high-density lipoprotein-cholesterol; LDL-C, low-density lipoprotein cholesterol*.

* *The P for trend value represent overall differences across groups, as determined by one-way analysis of variance test for continuous variables and Pearson's chi-squared test for categorical variables*.

### Risk of CVD According to the LDL-C Categories

**Figure 1:**
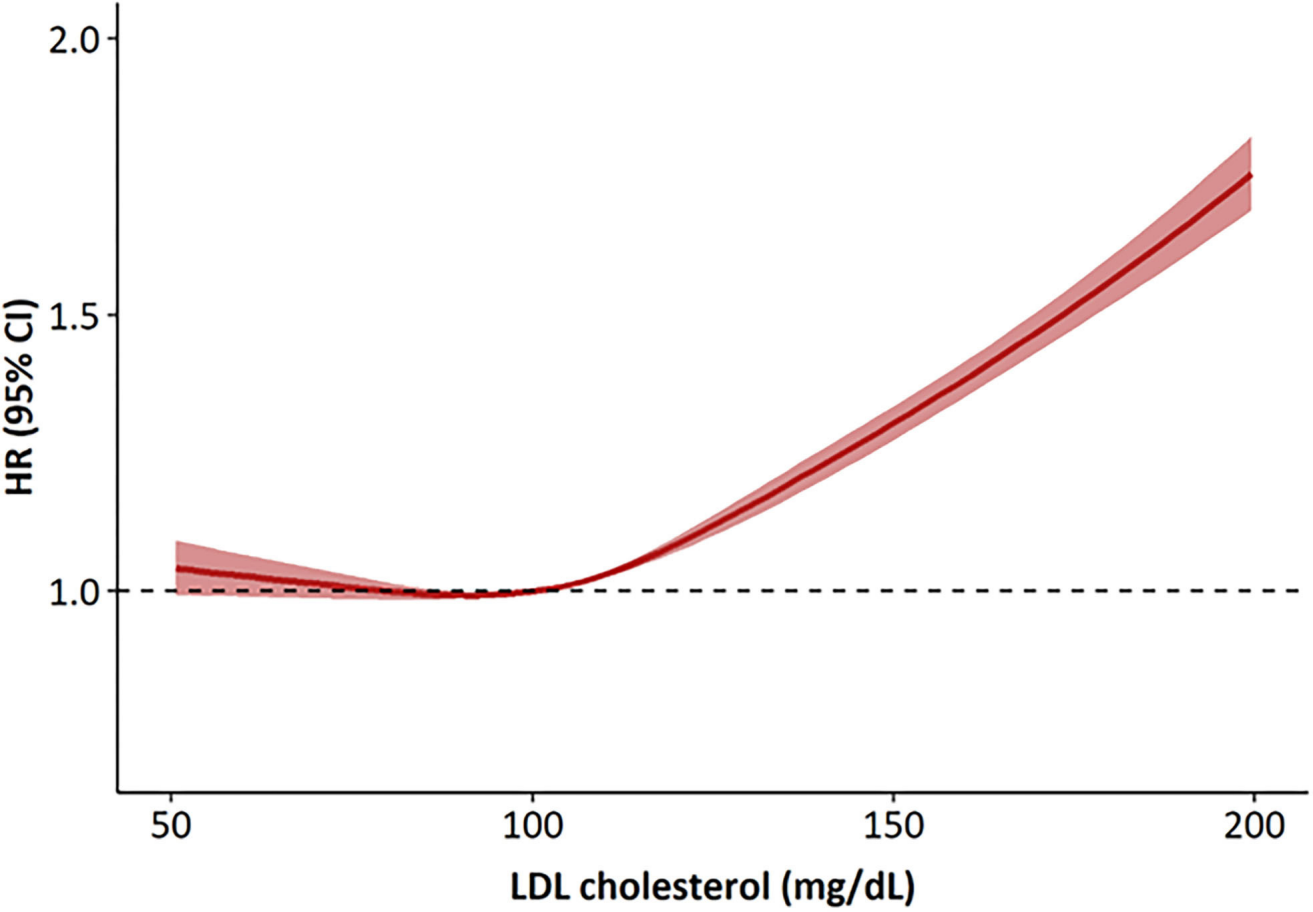
Hazard ratios for cardiovascular disease according to LDL cholesterol levels at baseline. The hazard ratios were calculated by Cox models after adjusting for age, sex, body mass index, smoking status, alcohol consumption, regular exercise, income and the presence of hypertension. HR, hazard ratios.

During the median follow-up of about 6 years, there were 51,961 incident CVD events that included 17,392 cases of MI, 33,779 cases of ischemic stroke, and 2,039 cardiovascular deaths. There was a linear relationship between the LDL-C levels and CVD risk in the study population (Figure 1). The number of events, incidence, and HRs for CVD increased significantly in the higher LDL-C categories (Table 2). Using an LDL-C level of 70–99 mg/dL as the reference group, an LDL-C level of ≥100 mg/dL was associated with a significantly higher risk of CVD. In the multivariable analysis, the adjusted HRs (95% CIs) for CVD in the <70, 100–129, 130–159, 160–189, and ≥190 mg/dL LDL-C categories were 1.02 (0.97–1.07), 1.09 (1.07–1.12), 1.30 (1.26–1.33), 1.51 (1.46–1.56), and 2.01 (1.89–2.14), respectively, compared with the 70–99 mg/dL LDL-C category. An LDL-C level of ≥100 mg/dL was associated with a significantly greater risk of CVD in non-statin users. The risk of CVD increased linearly from an LDL-C level ≥130 mg/dL in statin users compared to non-statin users. When this association was stratified by the type of cardiovascular event, LDL-C levels ≥100 mg/dL in non-statin users and ≥130 mg/dL in statin users were significantly associated with a higher risk of MI (Additional File 2). LDL-C levels ≥100 mg/dL in non-statin users and ≥190 mg/dL in statin users were correlated with a significantly higher risk of ischemic stroke. LDL-C levels ≥130 mg/dL in non-statin users and ≥190 mg/dL in statin users were associated with a significantly higher risk of cardiovascular deaths.

**Table 2:** Risk of primary outcome according to the baseline LDL-C levels excluding subjects who died within 2 years of follow-up.

**Statin use**	**LDL-C (mg/dL)**	**Person-years**	**Number of events**	**Incident rate**	**Age-adjusted**	**Multivariable adjusted**
				**(10,000 person years)**	**HR (95% CI)**	**HR (95% CI) #**
Overall	<70 mg/dL	1,278,498	2,079	16.26	1.1 (1.05–1.15)	1.02 (0.97–1.07)
	70–99 mg/dL	7,753,135	10,189	13.14	1.00 (Reference)	1.00 (Reference)
	100–129 mg/dL	13,934,274	19,674	14.12	1.07 (1.05–1.10)	1.09 (1.07–1.12)
	130–159 mg/dL	8,420,637	14,410	17.11	1.28 (1.25–1.31)	1.30 (1.26–1.33)
	160–189 mg/dL	2,241,436	4,467	19.93	1.50 (1.45–1.56)	1.51 (1.46–1.56)
	≥ 190 mg/dL	427,148	1,142	26.74	2.07 (1.95–2.20)	2.01 (1.89–2.14)
Statin non-user	<70 mg/dL	1,011,432	1,381	13.65	1.07 (1.01–1.14)	1.01 (0.95–1.07)
	70–99 mg/dL	6,788,255	7,876	11.60	1.00 (Reference)	1.00 (Reference)
	100–129 mg/dL	12,303,761	16,121	13.10	1.10 (1.08–1.13)	1.11 (1.08–1.15)
	130–159 mg/dL	6,679,456	10,924	16.35	1.36 (1.32–1.40)	1.37 (1.33–1.41)
	160–189 mg/dL	1,310,427	2,613	19.94	1.69 (1.62–1.77)	1.69 (1.61–1.76)
	≥190 mg/dL	170,941	505	29.54	2.58 (2.36–2.83)	2.47 (2.26–2.70)
Statin user	<70 mg/dL	267,066	698	26.14	1.03 (0.94–1.12)	0.99 (0.91–1.08)
	70–99 mg/dL	964,879	2,313	23.97	1.00 (Reference)	1.00 (Reference)
	100–129 mg/dL	1,630,513	3,553	21.79	0.99 (0.94–1.04)	1.02 (0.97–1.07)
	130–159 mg/dL	1,741,181	3,486	20.02	0.99 (0.94–1.04)	1.06 (1.00–1.11)
	160–189 mg/dL	931,009	1,854	19.91	1.04 (0.98–1.10)	1.16 (1.09–1.24)
	≥190 mg/dL	256,206	637	24.86	1.36 (1.25–1.49)	1.51 (1.38–1.66)

*# adjusted for age, sex, body mass index, smoking status, alcohol consumption, regular exercise, income and hypertension*.

*LDL-C, low lipoprotein cholesterol; HR, hazard ratios*.

### Risk of CVD According to the LDL-C Categories Within the FPG Strata

**Figure 2:**
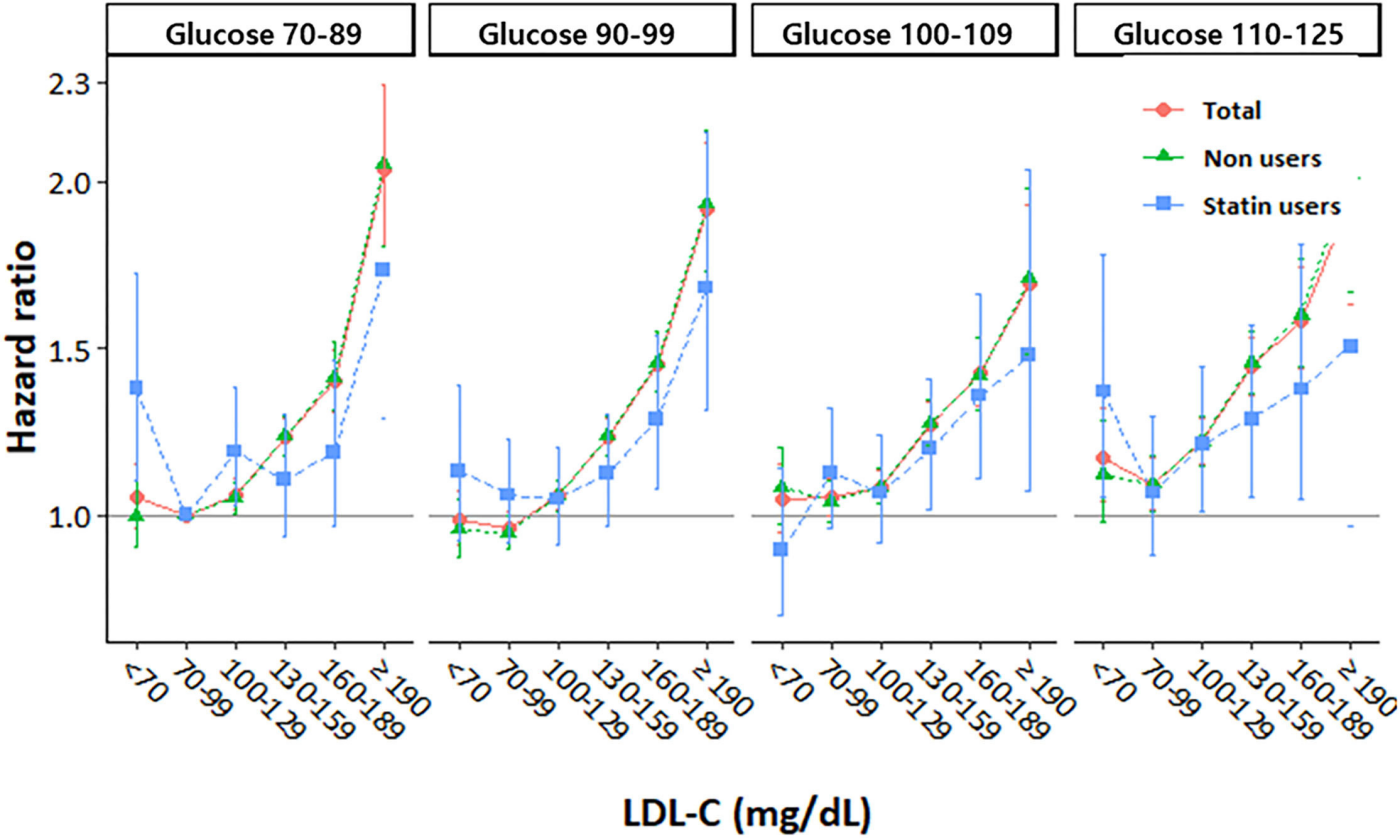
Hazard ratios for cardiovascular disease according to LDL cholesterol levels stratified by fasting glucose level at baseline.

We also analyzed the incidence values and HRs of CVD according to the LDL-C categories and stratified based on the FPG levels (70–90, 90–99, 100–109, and 110–125 mg/dL) (Table 3 and Figure 2). In all the FPG categories, we observed that the risk of CVD gradually increased as the LDL-C levels increased. Using the FPG category 70–99 mg/dL and LDL-C category 70–99 mg/dL as references, the CVD risk in the higher LDL-C group increased with worsening glycemic status. Moreover, even in participants with lower LDL-C levels, all those with FPG levels ≥110 mg/dL had a significantly higher risk of CVD than participants in the reference group (Table 3). However, the higher risk of CVD in those with high LDL-C and high FPG levels was more attenuated in statin users than in non-statin users (Figure 2).

**Table 3:** Risk of primary outcome according to the baseline LDL-C levels excluding subjects who died within 2 years of follow-up stratified by fasting glucose level at baseline.

**Glucose (mg/dL)**	**LDL-C (mg/dL)**	**Person-years**	**Number of events**	**Incident rate**	**Age-adjusted HR**	**Multivariable adjusted**
				**(10,000 person years)**	**(95% CI)**	**HR (95% CI)#**
70–90 mg/dL	<70 mg/dL	421783.9	568	13.47	1.20 (1.10–1.31)	1.05 (0.96–1.15)
	70–99 mg/dL	2891630.2	3,185	11.01	1.00 (Reference)	1.00 (Reference)
	100–129 mg/dL	4877874.2	5,948	12.19	1.03 (0.99–1.08)	1.06 (1.02–1.11)
	130–159 mg/dL	2,664,123	4,067	15.27	1.19 (1.14–1.25)	1.23 (1.18–1.29)
	160–189 mg/dL	647856.6	1,165	17.98	1.33 (1.25–1.43)	1.40 (1.31–1.50)
	≥ 190 mg/dL	117293.0	310	26.43	1.94 (1.73–2.18)	2.03 (1.81–2.29)
90–99 mg/dL	<70 mg/dL	469637.3	716	15.25	1.23 (1.14–1.34)	0.99 (0.91–1.07)
	70–99 mg/dL	3000266.7	3,759	12.53	1.04 (1.00–1.09)	0.96 (0.92–1.01)
	100–129 mg/dL	5609484.6	7,685	13.70	1.09 (1.05–1.14)	1.06 (1.02–1.10)
	130–159 mg/dL	3461224.7	5,689	16.44	1.24 (1.19–1.29)	1.23 (1.18–1.29)
	160–189 mg/dL	921301.7	1,806	19.60	1.41 (1.34–1.50)	1.45 (1.37–1.54)
	≥ 190 mg/dL	172577.0	457	26.48	1.85 (1.68–2.04)	1.92 (1.74–2.12)
100–109 mg/dL	<70 mg/dL	257539.9	484	18.79	1.42 (1.29–1.56)	1.05 (0.95–1.15)
	70–99 mg/dL	1328820.6	2,189	16.47	1.26 (1.20–1.33)	1.05 (1.00–1.11)
	100–129 mg/dL	2503745.5	4,074	16.27	1.23 (1.17–1.29)	1.09 (1.04–1.14)
	130–159 mg/dL	1656633.4	3,134	18.92	1.38 (1.31–1.45)	1.27 (1.21–1.34)
	160–189 mg/dL	478705.6	1,004	20.97	1.48 (1.38–1.59)	1.43 (1.33–1.53)
	≥ 190 mg/dL	95308.2	244	25.60	1.77 (1.56–2.02)	1.69 (1.48–1.93)
110–125 mg/dL	<70 mg/dL	129537.5	311	24.01	1.73 (1.54–1.94)	1.17 (1.04–1.32)
	70–99 mg/dL	532416.8	1,056	19.83	1.43 (1.33–1.53)	1.09 (1.02–1.17)
	100–129 mg/dL	943169.8	1,967	20.86	1.49 (1.41–1.58)	1.22 (1.15–1.29)
	130–159 mg/dL	638655.6	1,520	23.80	1.68 (1.58–1.79)	1.44 (1.36–1.54)
	160–189 mg/dL	193572.2	492	25.42	1.75 (1.59–1.93)	1.58 (1.44–1.74)
	≥ 190 mg/dL	41969.3	131	31.21	2.10 (1.77–2.51)	1.95 (1.63–2.32)

*# adjusted for age, sex, body mass index, smoking status, alcohol consumption, regular exercise, income and the presence of hypertension*.

*LDL-C, low lipoprotein cholesterol; HR, hazard ratios*.

## Discussion

We evaluated the risk of incident CVD according to the LDL-C and FPG levels in participants without DM. We observed significant positive associations between the increased risk of CVD and high LDL-C levels in participants without DM, and the risk of CVD increased in participants without DM who had LDL-C levels ≥100 mg/dL. We demonstrated that elevations in the FPG levels, even in the same LDL-C category, were associated with an increased risk of CVD. The risk of CVD increased more significantly in participants with FPG levels ≥110 mg/dL than in those in the other FPG categories. These findings suggested that elevations in the FPG and LDL-C levels independently contributed to the increased risk of CVD in participants without DM. To the best of our knowledge, this was the first nationwide study to investigate the optimal ranges of LDL-C that were associated with the lowest risk of CVD in East Asian adults without DM.

Previous epidemiological investigations have consistently demonstrated a strong positive, continuous, independent, and graded relationship between LDL-C levels and the incidence of CVD (11, 12). Furthermore, recent meta-analyses of Mendelian randomization studies involving over 300,000 participants and 80,000 CVD cases provided convincing evidence regarding the causal correlation between LDL-C levels and the risk of ASCVD. Moreover, they showed that the causal impact of LDL-C levels on ASCVD might essentially be independent of the mechanism by which LDL-C levels are “lowered” (13). Therefore, most international guidelines suggest strategies for managing LDL-C levels and setting LDL-C targets for the primary prevention of CVD. These guidelines consider DM to be a major risk factor for CVD and recommend the use of statins, regardless of the LDL-C levels, for the prevention of CVD in patients with DM (14–16). However, the implementation of guidelines among members of the general population, especially among those without DM, has been a challenge for a long time. The American College of Cardiology/American Heart Association (ACC/AHA) guidelines removed specific target LDL-C levels since 2013 and emphasized a strategy of fixed-dose statin therapy based on cardiovascular risk in individuals without DM (14). However, calculating the ASCVD risk is challenging due to the lack of time and complexities in clinical calculations. Moreover, this ASCVD risk calculator was designed based on data mainly from populations other than those from East Asia. Considering the established strong graded relationship between LDL-C levels and incident CVD, determining the optimal LDL-C range for the primary prevention of CVD in individuals without DM is needed in the Asian population.

The present study found that among participants without DM and a history of CVD, there was an increased risk of CVD in those with LDL-C levels ≥100 mg/dL This cut-off value was in line with that recommended by several international guidelines. The 2019 European Society of Cardiology/European Atherosclerotic Society guidelines suggest an LDL-C goal of <116 mg/dL for the primary prevention of CVD in individuals with a low risk of CVD (16). However, guidelines for dyslipidemia management in Korea suggest an LDL-C goal of <130 mg/dL in individuals without DM and a moderate CVD risk, and <160 mg/dL in individuals without DM and a low CVD risk (15). The suggested target LDL-C levels according to the Korean guidelines in individuals without DM were significantly higher than those in our study (LDL-C levels <100 mg/dL). However, the Korean dyslipidemia guidelines were made based on data from other countries. Further prospective randomized controlled studies are warranted to determine the optimal LDL-C levels for the initiation of pharmacological intervention for the primary prevention of CVD in Korean adults without DM.

In the current study, we observed a graded positive trend for CVD risk starting from an LDL-C level of 100 mg/dL, which increased for the higher LDL-C categories among participants who were non-statin users. The risk of CVD was higher whose LDL-C levels were ≥130 mg/dL in statin users than in those in the LDL-C reference group. From these results, we suggest that the uptitration of statins may be considered if LDL-C levels are ≥130 mg/dL during statin treatment, with consideration of the CVD risk in individuals without DM. Furthermore, the risk of CVD was relatively more attenuated in statin users than in non-statin users, even in the same LDL-C categories. The attenuated CVD risk in statin users was prominent in those in the higher LDL-C and FPG categories. It demonstrated the benefit of using statin for the primary prevention of CVD, regardless of the LDL-C levels in individuals without DM. This finding was consistent with those of previous primary prevention trials that demonstrated the benefits of statin therapy (17). These studies showed that statins can reduce CVD risk through pleiotropic effects, including the inhibition of inflammation (6, 18).

We found that modestly elevated FPG levels (110–125 mg/dL), even it is not suitable level for diagnosing diabetes, were independently associated with a higher risk of CVD compared to the reference groups (FPG level of 70–99 mg/dL and LDL-C level of 70–99 mg/dL). A higher risk of CVD in participants with modestly elevated FPG levels was still observed even when their LDL-C levels were low (70 mg/dL). This suggested that well-controlled LDL-C levels might not be protective against a higher CVD risk in individuals with modestly elevated FPG levels. Moreover, we observed that the combination of higher FPG and LDL-C levels synergistically elevated the risk of CVD in participants without DM. This finding was consistent with the biological synergistic interaction between glucose and cholesterol levels reported in previous studies (19, 20). There are some possible biological mechanisms that support the interaction between glucose and LDL-C. It is widely believed that the oxidation of LDL-C plays an important role in atherogenesis, and excess circulating glucose levels might facilitate cholesterol peroxidation (21). It was found that DM might be related to oxidative stress, which is linked to atherogenesis (22–24). This biological interaction indicates that optimal LDL-C goals might differ according to glucose levels, which might be clinically significant. Our findings warrant a clinical trial to determine whether using glucose levels to advise about cholesterol control would improve outcomes. Our findings indicated that the target LDL-C goal for the primary prevention of CVD should be lower, and more aggressive statin use may be considered in individuals with FPG levels of 110–125 mg/dL, similar to those with DM.

Our study had several limitations. First, as the NHIS database relies on the issuance of a diagnostic code for CVD by physicians, there might have been a risk of misdiagnosis, which might have contributed to the underestimation or overestimation of the prevalence of CVD. Second, day-to-day variabilities might have influenced the findings due to laboratory errors or biological variations, as we used the results of a single LDL-C and FPG test in the analyses. Additionally, as we could not directly measure LDL-C levels and used the Friedewald formula instead, it might have led to the underestimation of the LDL-C levels. Third, there was lack of data on antidyslipidemic medication use among our participants during the follow-up period. Over time, cholesterol levels can increase, which could have led to statin use even among non-users, thereby mitigating the observed risk of CVD. We did not perform time-varying Cox regression considering these factors and this is limitation of our study. Fourth, we did not obtain data on changes in medications or interventions during the follow-up period. Furthermore, we did not account for many confounders in our study, such as genetic factors, medication use, and socioeconomic status, which might have influenced our results. Fifth, we could not calculate the 10 year risk of ASCVD due to lack of data and consider individual cardiovascular risks in our analysis. Finally, as the present study only included the Korean population, our findings could not be generalized to other ethnicities. However, the major strengths of the current study were its large sample size, with approximately 4,000,000 relatively healthy general populations, and use of longitudinal data. Thus, our results reflect “real-world” evidence on the association of LDL-C levels with CVD risk in individuals without DM on a national scale. Finally, we used only fasting glucose level as a surrogate marker for glycemic status because HbA1c measurement was not included the national health screening program in our country during the study period.

## Conclusions

In conclusion, we demonstrated that LDL-C levels ≥100 mg/dL increased the risk of CVD in individuals without DM. Furthermore, we found that a glucose–cholesterol interaction magnified the CVD risk even in those without DM, and the CVD risk was more attenuated in statin users than in non-statin users. We observed a graded positive trend in CVD risk starting from an LDL-C level of 100 mg/dL, and this risk increased in the higher LDL-C categories. Thus, more active initiation of statin treatment for the primary prevention of CVD in Korean adults without DM can be considered when their LDL-C levels are ≥100 mg/dL. Moreover, given that participants with modestly elevated FPG levels (especially FPG levels ≥110 mg/dL) had a high risk of CVD after adjusting for confounders, even those with lower LDL-C levels, earlier initiation of statin treatment for the primary prevention of CVD may be considered for these participants. Further large-scale, long-term, follow-up randomized control studies are warranted to clearly determine the optimal LDL-C target for the primary prevention of CVD in individuals without DM.

## Data Availability Statement

The datasets presented in this article are not readily available because the datasets generated and analyzed during the current study are not publicly available due to rule of Korea National health insurance system. Requests to access the datasets should be directed to kimjang713@gmail.com.

## Ethics Statement

This study was approved by Institutional Review Board of Yonsei University Wonju College of Medicine, Republic of Korea (no. CR318356). The patients/participants provided their written informed consent to participate in this study.

## Author Contributions

JH and SP conceived the study concept and design. T-HG and DK acquired data and performed statistical analyses. JH and SP wrote the first draft and conducted the literature search. JH, S-HL, and J-YK analyzed and interpreted data. J-YK is the guarantor of this work and, as such, had full access to all the data in the study and takes responsibility for the integrity of the data, and the accuracy of the data analysis. All authors contributed to critical revision of the manuscript, read, and approved the final submitted version of the manuscript.

## Funding

This study was supported by a National Research Foundation of Korea grant funded by the Korean government (No. NRF-2019R1G1A109408) and Korean Society of Lipid and Atherosclerosis.

## Conflict of Interest

The authors declare that the research was conducted in the absence of any commercial or financial relationships that could be construed as a potential conflict of interest.

## Publisher's Note

All claims expressed in this article are solely those of the authors and do not necessarily represent those of their affiliated organizations, or those of the publisher, the editors and the reviewers. Any product that may be evaluated in this article, or claim that may be made by its manufacturer, is not guaranteed or endorsed by the publisher.
