# Supplementary material for: Optimal Low-Density Lipoprotein Cholesterol Levels in Adults Without Diabetes Mellitus: A Nationwide Population-Based Study Including More Than 4 Million Individuals From South Korea
Source: Front Cardiovasc Med. 2022 Jan 20;8:812416. doi: 10.3389/fcvm.2021.812416 (PMC8811156; doi:10.3389/fcvm.2021.812416)
Supplement: Supplementary file 1 [file Data_Sheet_1.PDF]

Supplementary Figure 1. Study population

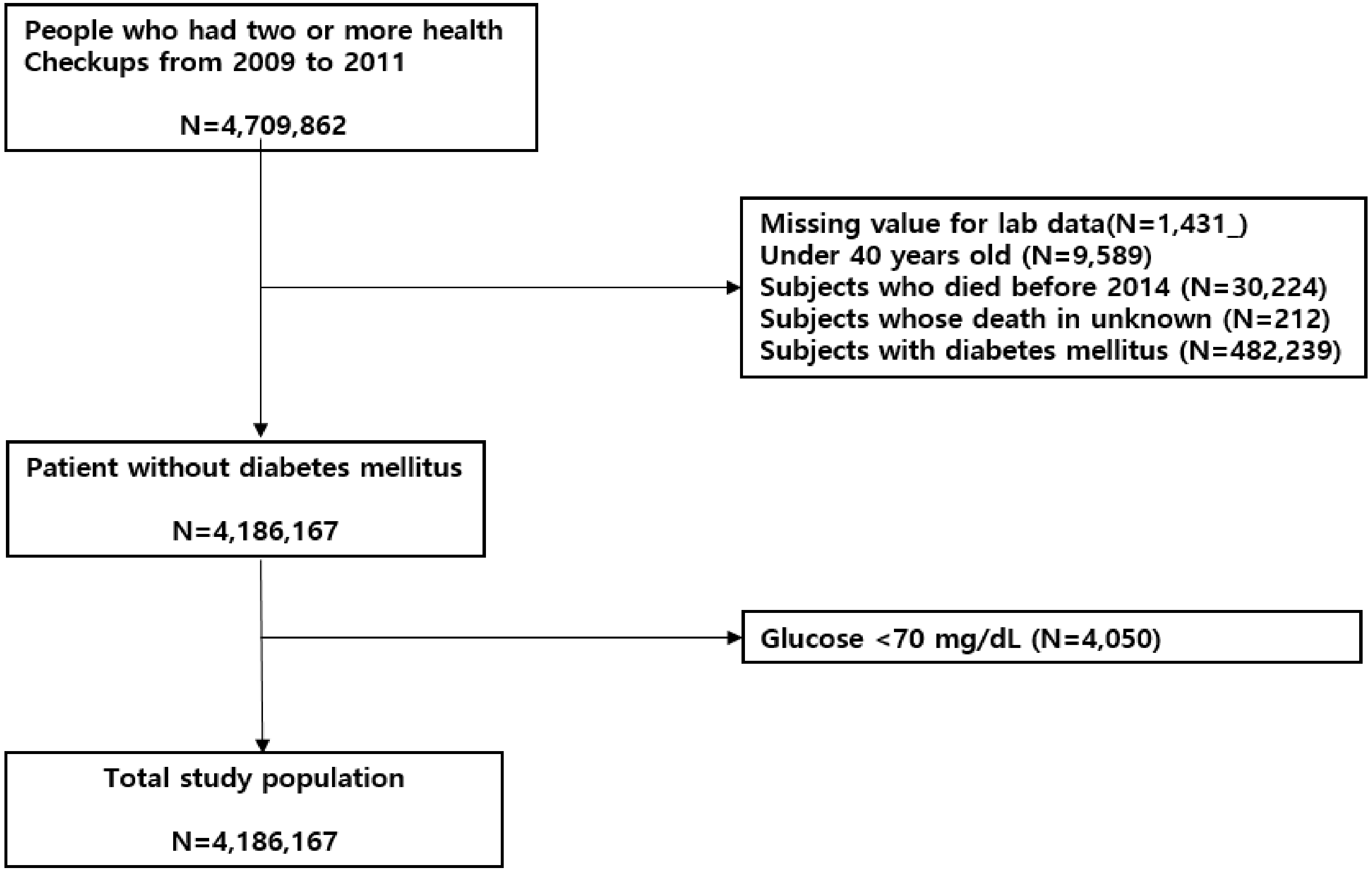

Supplementary Table 2. Risk of cardiovascular disease according to baseline LDL-C levels excluding subjects who died within 2 yers of follow-up

(a) Risk of MI according to baseline LDL-C levels excluding subjects who died within 2 yers of follow-up

Statin use	LDL-C (mg/dL)	Person-years	Number of events	Incident rate (10,000 person years)	Age-adjusted HR (95% CI)	Multivariable adjusted HR# (95% CI)
Overall	<70 mg/dL	1280386	555	4.33	1.02 (0.94 - 1.12)	0.94 (0.85 - 1.02)
	70-99 mg/dL	7763341	2818	3.63	1.00 (Reference)	1.00 (Reference)
	100-129 mg/dL	13954030	6127	4.39	1.24 (1.18 - 1.29)	1.25 (1.2 - 1.31)
	130-159 mg/dL	8434176	5441	6.45	1.85 (1.77 - 1.93)	1.81 (1.73 - 1.89)
	160-189 mg/dL	2245311	1931	8.60	2.58 (2.44 - 2.74)	2.40 (2.26 - 2.54)
	≥ 190 mg/dL	428088	520	12.15	3.91 (3.56 - 4.29)	3.35 (3.04 - 3.69)
Non-statin user	<70 mg/dL	1012694	293	2.89	0.86 (0.76 - 0.98)	0.82 (0.73 - 0.93)
	70-99 mg/dL	6796195	1997	2.94	1.00 (Reference)	1.00 (Reference)
	100-129 mg/dL	12320056	4839	3.93	1.35 (1.28 - 1.42)	1.33 (1.27 - 1.41)
	130-159 mg/dL	6689616	4122	6.16	2.13 (2.02 - 2.24)	2.07 (1.96 - 2.18)
	160-189 mg/dL	1312618	1176	8.96	3.20 (2.98 - 3.44)	3.05 (2.83 - 3.28)
	≥ 190 mg/dL	171350	229	13.36	5.02 (4.38 - 5.76)	4.52 (3.94 - 5.19)
Statin user	<70 mg/dL	267692	262	9.79	1.02 (0.88 - 1.17)	0.99 (0.86 - 1.14)
	70-99 mg/dL	967147	821	8.49	1.00 (Reference)	1.00 (Reference)
	100-129 mg/dL	1633974	1288	7.88	1.04 (0.95 - 1.13)	1.06 (0.97 - 1.16)
	130-159 mg/dL	1744560	1319	7.56	1.10 (1.00 - 1.20)	1.16 (1.06 - 1.27)
	160-189 mg/dL	932693	755	8.09	1.25 (1.13 - 1.38)	1.39 (1.26 - 1.54)
	≥ 190 mg/dL	256739	291	11.33	1.86 (1.63 - 2.13)	2.02 (1.76 - 2.32)

#Adjusted for age, body mass index, smoking status, alcohol consumption, regular exercise, income and hypertension

LDL-C, low lipoprotein cholesterol; HR, hazard ratios

(b) Risk of ischemic stroke outcome according to baseline LDL-C levels excluding subjects who died within 2 yers of follow-up

Statin use	LDL-C (mg/dL)	Person-years	Number of events	Incident rate (10,000 person years)	Age-adjusted HR (95% CI)	Multivariable ajdusted HR (95% CI)
overall	<70 mg/dL	1279293	1484	11.60	1.2 (1.14 - 1.27)	1.07 (1.01 - 1.13)
	70-99 mg/dL	7757907	7207	9.29	1.00 (Reference)	1.00 (Reference)
	100-129 mg/dL	13945626	13274	9.52	0.98 (0.95 – 1.00)	1.02 (0.99 - 1.05)
	130-159 mg/dL	8431785	8737	10.36	0.99 (0.96 - 1.02)	1.07 (1.04 - 1.10)
	160-189 mg/dL	2245476	2474	11.02	1.01 (0.96 - 1.05)	1.12 (1.07 - 1.18)
	≥ 190 mg/dL	428284	603	14.08	1.25 (1.15 - 1.36)	1.42 (1.30 - 1.54)
Non-statin user	<70 mg/dL	1011686	1051	10.39	1.15 (1.07 - 1.22)	1.07 (1 - 1.14)
	70-99 mg/dL	6791357	5748	8.46	1.00 (Reference)	1.00 (Reference)
	100-129 mg/dL	12312343	11056	8.98	1.02 (0.99 - 1.05)	1.04 (1.00 - 1.07)
	130-159 mg/dL	6687745	6625	9.91	1.09 (1.05 - 1.13)	1.11 (1.07 - 1.15)
	160-189 mg/dL	1312853	1401	10.67	1.19 (1.12 - 1.26)	1.21 (1.14 - 1.28)
	≥ 190 mg/dL	171430	263	15.34	1.73 (1.53 - 1.96)	1.69 (1.50 - 1.92)
Statin user	<70 mg/dL	267607	433	16.18	1.06 (0.95 - 1.18)	1.01 (0.91 - 1.13)
	70-99 mg/dL	966550	1459	15.09	1.00 (Reference)	1.00 (Reference)
	100-129 mg/dL	1633282	2218	13.58	0.96 (0.90 - 1.03)	0.99 (0.93 - 1.06)
	130-159 mg/dL	1744040	2112	12.11	0.92 (0.86 - 0.99)	0.99 (0.92 - 1.06)
	160-189 mg/dL	932623	1073	11.51	0.92 (0.85 – 1.00)	1.03 (0.95 - 1.12)
	≥ 190 mg/dL	256853	340	13.24	1.11 (0.98 - 1.25)	1.24 (1.10 - 1.40)

#Adjusted for age, body mass index, smoking status, alcohol consumption, regular exercise, income and hypertension

LDL-C, low lipoprotein cholesterol; HR, hazard ratios

(c) Risk of CVD death according to baseline LDL-C levels excluding subjects who died within 2 yers of follow-up

Statin use	LDL-C (mg/dL)	Person-years	Number of events	Incident rate (10,000 person years)	Age-adjusted HR (95% CI)	Multivariable adjusted HR (95% CI)
overall	<70 mg/dL	1281946	111	0.87	1.59 (1.29 - 1.96)	1.30 (1.05 - 1.60)
	70-99 mg/dL	7770519	402	0.52	1.00 (Reference)	1.00 (Reference)
	100-129 mg/dL	13968825	754	0.54	0.99 (0.88 - 1.12)	1.08 (0.96 - 1.22)
	130-159 mg/dL	8447325	542	0.64	1.08 (0.95 - 1.23)	1.27 (1.12 - 1.45)
	160-189 mg/dL	2249893	169	0.75	1.20 (1.00 - 1.44)	1.55 (1.29 - 1.86)
	≥ 190 mg/dL	429364	61	1.42	2.19 (1.68 - 2.87)	2.92 (2.22 - 3.85)
Non-statin user	<70 mg/dL	1013625	88	0.87	1.61 (1.27 - 2.04)	1.46 (1.15 - 1.85)
	70-99 mg/dL	6801463	314	0.46	1.00 (Reference)	1.00 (Reference)
	100-129 mg/dL	12331750	609	0.49	1.08 (0.94 - 1.23)	1.11 (0.97 - 1.28)
	130-159 mg/dL	6699606	413	0.62	1.36 (1.18 - 1.58)	1.41 (1.21 - 1.64)
	160-189 mg/dL	1315395	108	0.82	1.93 (1.55 - 2.40)	2.00 (1.60 - 2.50)
	≥ 190 mg/dL	171917	30	1.75	4.34 (2.98 - 6.32)	4.27 (2.93 - 6.23)
Statin user	<70 mg/dL	268321	23	0.86	0.87 (0.55 - 1.38)	0.81 (0.50 - 1.29)
	70-99 mg/dL	969056	88	0.91	1.00 (Reference)	1.00 (Reference)
	100-129 mg/dL	1637074	145	0.89	1.11 (0.85 - 1.45)	1.16 (0.89 - 1.52)
	130-159 mg/dL	1747718	129	0.74	1.05 (0.80 - 1.38)	1.15 (0.87 - 1.52)
	160-189 mg/dL	934498	61	0.65	1.02 (0.73 - 1.42)	1.20 (0.86 - 1.68)
	≥ 190 mg/dL	257448	31	1.20	2.05 (1.36 - 3.10)	2.34 (1.53 - 3.58)

#Adjusted for age, body mass index, smoking status, alcohol consumption, regular exercise, income and hypertension

LDL-C, low lipoprotein cholesterol; HR, hazard ratios
